# Randomised, phase III trial of epoetin-*β* to treat chemotherapy-induced anaemia according to the EU regulation

**DOI:** 10.1038/bjc.2011.395

**Published:** 2011-09-29

**Authors:** Y Fujisaka, T Sugiyama, H Saito, S Nagase, S Kudoh, M Endo, H Sakai, Y Ohashi, N Saijo

**Affiliations:** 1 Respiratory Medicine, Osaka Medical College Hospital, Osaka, Japan;; 2 Department of Obstetrics and Gynecology, Iwate Medical University School of Medicine, Iwate, Japan; 3 Department of Respiratory Medicine, Aichi Cancer Center Aichi Hospital, Aichi, Japan; 4 Department of Thoracic Surgery, Tokyo Medical University, Tokyo, Japan; 5 Department of Respiratory Medicine, Osaka City University Hospital, Osaka, Japan; 6 Division of Thoracic Oncology, Shizuoka Cancer Center, Shizuoka, Japan; 7 Department of Thoracic Oncology, Saitama Cancer Center, Saitama, Japan; 8 Department of Biostatistics, School of Public Health, University of Tokyo, Tokyo, Japan; 9 Department of Medical Oncology, Kinki University Faculty of Medicine, 377-2, Ohno-higashi, Osakasayama, Osaka 589-8511, Japan

**Keywords:** chemotherapy-induced anaemia, erythropoietin, survival

## Abstract

**Background:**

Erythropoietin-stimulating agents (ESAs) effectively decrease the transfusion requirements of patients with chemotherapy-induced anaemia (CIA). Recent studies indicate that ESAs increase mortality and accelerate tumour progression. The studies also identify a 1.6-fold increased risk of venous thromboembolism. The ESA labelling was thus revised in Europe and the United States in 2008. This is the first randomised, phase III trial evaluating the efficacy and safety of epoetin-*β* (EPO), an ESA, dosed in accordance with the revised labelling, which specifies that ESAs should be administered to CIA patients with a haemoglobin level of ⩽10 g dl^–1^ and that a sustained haemoglobin level of >12 g dl^–1^ should be avoided.

**Methods:**

A total of 186 CIA patients (8.0 g dl^–1^⩽ haemoglobin ⩽10.0 g dl^–1^) with lung or gynaecological cancer were randomised to receive EPO 36 000 IU or placebo weekly for 12 weeks.

**Results:**

The proportion of patients receiving transfusions or with haemoglobin <8.0 g dl^–1^ between week 5 and the end of the treatment period as the primary end point was significantly lower in the EPO group (*n*=89) than in the placebo group (*n*=92; 10.0% *vs* 56.4%, *P*<0.001). The proportion receiving transfusions was significantly lower in the EPO group (4.5% *vs* 19.6%, *P*=0.002). Changes in quality of life were not different. No significant differences in adverse events – for example, the incidence of thromboembolic events was 1.1% for each group – or the 1-year overall survival were observed between groups.

**Conclusion:**

Weekly EPO administered according to the revised labelling approved by the European Medicines Agency is effective and well tolerated for CIA treatment. Further investigations are needed on the effect of ESAs on mortality.

Anaemia is a common adverse event in cancer patients receiving chemotherapy, particularly in patients with lung and gynaecological cancers (Ludwig *et al*, 2004; Ohe *et al*, 2007; Katsumata *et al*, 2009). Several of the symptoms associated with anaemia, such as fatigue, syncope, palpitations and dyspnoea, reduce patient activity and have a profound effect on the quality of life (QOL) (Bokemeyer *et al*, 2007). Red blood cell (RBC) transfusion is one of the available treatments for anaemia. However, RBC transfusion is associated with a risk of volume overload, infection of unknown virus and transfusion reactions. And in Japan, blood transfusion therapy is problematic because of an increasing demand for blood products and a scarcity of blood supply arising from the declining birth rate and ageing population.

In Europe and the United States, erythropoiesis-stimulating agents (ESAs) have been used since 1993 for the treatment of chemotherapy-induced anaemia (CIA). The ESAs increase haemoglobin levels and reduce the need for RBC transfusion (Littlewood *et al*, 2001; Österborg *et al*, 2002). Since 2003, several studies have suggested that ESAs are associated with increased mortality and/or tumour progression in cancer patients when administered with a target haemoglobin level of >12 g dl^–1^ (Hedenus *et al*, 2003; Henke *et al*, 2003; Leyland-Jones *et al*, 2005; Overgaard *et al*, 2007; Wright *et al*, 2007; Smith *et al*, 2008; Thomas *et al*, 2008). Accordingly, the risks of ESAs have been investigated by regulatory authorities (Juneja *et al*, 2008) and, in response to these investigations, the labelling of ESAs in Europe and the United States was revised in 2008. A recent meta-analysis of ESAs has suggested that the increase in mortality in ESA-treated cancer patients undergoing chemotherapy is less pronounced than in those patients undergoing other anticancer treatments such as radiotherapy or no anticancer treatment (Bohlius *et al*, 2009a, 2009b). Similarly, another meta-analysis indicated that when used within current European Organisation for Research and Treatment of Cancer (EORTC) treatment guidelines, the ESA epoetin-*β* (EPO) had no negative impact on survival and tumour progression (Aapro *et al*, 2008a). However, the risks of ESAs have also been shown to be independent of haemoglobin levels and dosing (Bennett *et al*, 2008; Bohlius *et al*, 2009a, 2009b), and these meta-analyses were not able to verify that the risks of ESAs were completely eradicated by adherence to the new labelling.

The purposes of this study were to evaluate the efficacy and safety of EPO for the treatment of CIA with a dosing strategy according to the current labelling approved by the European Medicines Agency (EMA) (inclusion haemoglobin level criteria ⩽10 g dl^–1^, ceiling haemoglobin level=12 g dl^–1^). We previously conducted a dose-finding study of once-weekly EPO in CIA patients with malignant lymphoma or lung cancer, and recommended a weekly dose of 36 000 IU based on our results (Morishima *et al*, 2006).

## Patients and methods

### Patient eligibility

Inclusion criteria were as follows: (1) lung or gynaecological cancer; (2) receiving platinum-based chemotherapy and expected to receive at least two additional cycles of chemotherapy; (3) CIA (8.0 g dl^–1^⩽haemoglobin level ⩽10.0 g dl^–1^); (4) age between 20 and 79 years; (5) Eastern Cooperative Oncology Group performance status (PS) of 0–2; and (6) adequate hepatic and renal function.

Exclusion criteria included: (1) iron-deficiency anaemia (serum transferrin saturation (TSAT) <15% or mean corpuscular volume (MCV) <80 *μ*m^3^); (2) ESA therapy within 8 weeks or RBC transfusion within 4 weeks before the study; (3) surgery scheduled during the study period; (4) previous radiation therapy to the pelvis; (5) documented haemorrhagic lesions; (6) history of myocardial, pulmonary or cerebral infarction; (7) uncontrolled hypertension; (8) history of hypersensitivity to ESA; (9) serious drug allergy; and (10) tumour in the central nervous system.

### Study design and treatment

This multicentre, randomised, double-blind, placebo-controlled, phase III study was conducted at 37 sites in Japan. The protocol was approved by the institutional review board of the respective hospitals, and written informed consent was obtained from all patients who participated in the study. Patients were randomised 1 : 1 to receive EPO 36 000 IU or placebo subcutaneously once a week for up to 12 weeks. Epoetin-*β* and placebo were supplied by Chugai Pharmaceutical Co., Ltd (Tokyo, Japan). Participants in the study and investigators (outcome assessors) were blinded toward treatment allocation. Randomisation was conducted by a contract research organisation (CRO) that was independent from the investigators. The randomisation was carried out by a central registration system and was stratified by tumour type, PS, haemoglobin level and institution using a dynamic balancing method. The randomisation table was kept sealed and stored until a database lock by the CRO. Analysis methods were determined before the database lock.

If the haemoglobin level increased to >12.0 g dl^–1^ at any time during the study, administration was discontinued until the haemoglobin level decreased to ⩽11.0 g dl^–1^, and was then restarted at two-thirds of the previous dose (24 000 IU). If the planned cycle of chemotherapy was completed or discontinued, treatment was withheld at 6 weeks after day 1 of the final chemotherapy cycle. A daily dose of 100–200 mg elemental iron was administrated if TSAT fell to <15% or MCV fell to <80 *μ*m^3^. The RBC transfusion was allowed at the discretion of the investigator during the study.

### Evaluation of efficacy and safety

The primary end point of this study was the proportion of patients receiving RBC transfusion or with a haemoglobin level <8.0 g dl^–1^ between week 5 and the end of the treatment period (EOTP). The secondary end points were the proportion of patients receiving RBC transfusion between week 5 and the EOTP, change in haemoglobin level and QOL from baseline to the EOTP. QOL was evaluated using the Japanese Functional Assessment of Cancer Therapy-Anaemia (FACT-An) questionnaire (Yoshimura *et al*, 2004). In this study, the FACT-An total fatigue subscale, which consists of 13 fatigue-related questions, was the principal means of analysis. The FACT-An total fatigue subscale scores (FSS) range from 0 to 52, with higher scores indicating less fatigue.

Safety end points included adverse events, tumour progression and death (during the treatment phase and 1-year follow-up period). Adverse events were assessed according to the National Cancer Institute Common Toxicity Criteria, ver. 3, translated by the Japan Clinical Oncology Group. The presence of neutralising antibodies to EPO was assessed at baseline and the EOTP.

### Statistical analysis

The sample size of 160 patients (including an anticipated withdrawal rate of 40%, mainly because of completing or discontinuing the planned cycle of chemotherapy) was calculated to yield 80% power to significantly detect a 25% reduction (from 45 to 20%) in the primary end point, the proportion of patients receiving RBC transfusion or with a haemoglobin level <8.0 g dl^–1^ between week 5 and the EOTP. Statistical testing was conducted using a two-sided significance level of *P*=0.05. The study was not powered for QOL as a secondary efficacy end point. Patients who received at least one dose of the study drug comprised the safety population. For efficacy analysis, ineligible patients were excluded from the safety population, resulting in the full analysis set (FAS) population. The proportion of patients receiving RBC transfusion or with a haemoglobin level <8.0 g dl^–1^ was estimated by the Kaplan–Meier method. The requirement for RBC transfusion was compared using the *χ*^2^ method. Changes in the haemoglobin level and FSS between groups were compared using Student's *t*-test.

## Results

### Demographics and baseline characteristics

A total of 186 patients were enroled in the study between June and December 2008, and 181 (89 EPO and 92 placebo) of these were eligible for efficacy evaluation (the FAS population). Five patients were excluded because of discontinuation before the first dosing for the following reasons: withdrawal of patient consent (*n*=2), chemotherapy regimen cancelled (*n*=1), patient eligibility criteria violation (*n*=1) and a positive result in the skin test to EPO (*n*=1). In all, 51 (57%) patients in the EPO group and 55 (60%) in the placebo group completed 12 weeks of the study. Elemental iron was administrated in 40 patients (45%) in the EPO group and 32 (35%) in the placebo group. The demographics and baseline characteristics of the FAS population were well balanced (Table 1). The range of haemoglobin levels at screening was 8.0–10.0 g dl^–1^, whereas those at baseline (1–17 days after the screening) ranged from 7.2 to 11.4 g dl^–1^. The main chemotherapeutic regimen for both lung and gynaecological cancer was carboplatin-paclitaxel therapy.

### Transfusion-related and haemoglobin end points

**Figure 1:**
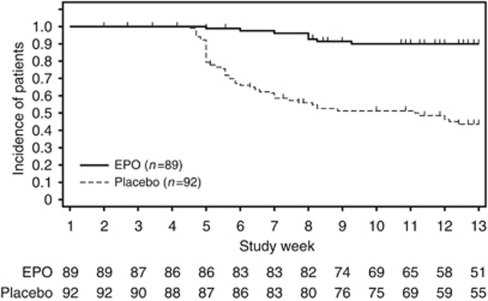
Time to RBC transfusion or haemoglobin level <8.0 g dl^–1^.

The proportion of patients receiving RBC transfusion or with a haemoglobin level <8.0 g dl^–1^ between week 5 and the EOTP was significantly lower in the EPO group than the placebo group (10.0% 95% confidence intervals (CIs) in the EPO group, 3.4–16.6 *vs* 56.4% 95% CI in the placebo group, 45.4–67.4%, *P*<0.001; Figure 1). Fewer patients received RBC transfusions between week 5 and the EOTP in the EPO group (4 of 89 patients, 4.5%) than in the placebo group (18 of 92 patients, 19.6%, *P*=0.002). The range of pretransfusion haemoglobin levels at the time of the first transfusion was 5.3–8.1 g dl^–1^.

**Figure 2:**
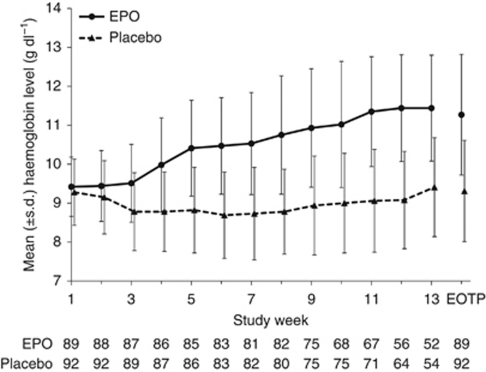
Change in haemoglobin level by treatment group. Abbreviation: EOTP=end of treatment period.

The mean change in haemoglobin level from baseline to the EOTP in the EPO group (1.9 g dl^–1^) was significantly higher than that in the placebo group (0.0 g dl^–1^, *P*<0.001). Figure 2 shows the mean changes in haemoglobin levels throughout the study in both groups. The mean nadir haemoglobin level between week 5 and the EOTP was 9.7 g dl^–1^ in the EPO group and 7.9 g dl^–1^ in the placebo group (*P*<0.001).

The percentage of patients with a haemoglobin level >12.0 g dl^–1^ after dosing, and whose administration was halted, was 50% in the EPO group and 2% in the placebo group.

### QOL

Overall compliance in terms of the percentage of patients who completed the FACT-An questionnaire was 98.3% (178 of 181) at baseline and 93.9% (170 of 181) at the end of the study. The mean baseline FSS was 35 points in the EPO group and 33 points in the placebo group. The mean changes in FSS from baseline to the EOTP in the EPO group were higher than in the placebo group, but these changes did not achieve statistical significance (0.30 *vs* −0.99, *P*=0.387).

### Safety

A total of 181 patients received study treatment and were included in the safety analysis. The overall incidence of adverse events was similar between the two groups (99% EPO and 100% placebo). There were 120 adverse events (in 37 patients) related to the study drug (adverse drug reactions) in the EPO group and 78 (in 28 patients) in the placebo group. Of these adverse drug reactions, constipation (6.7%), increased blood pressure (5.6%) and diarrhoea (5.6%) were reported by at least 5% of patients in the EPO group (Table 2). In all, 8 patients (14 events) in the EPO group and 17 patients (21 events) in the placebo group experienced serious adverse events. Of these, 5 events (acute respiratory distress syndrome, pneumonia, pulmonary embolism, neutropenia and thrombocytopenia) were considered to be related to EPO. As a thromboembolic event, one pulmonary embolism was observed in the EPO group. It was not associated with higher haemoglobin level (the haemoglobin level at the onset was 9.4 g dl^–1^). In the placebo group, haemorrhagic cerebral infarction (asymptomatic; no treatment was required) occurred in one patient. The proportion of patients who experienced tumour progression during the treatment period was similar in both groups (27.0% in the EPO group and 26.1% in the placebo group). No neutralising antibodies to EPO were detected.

### Survival

One patient in the EPO group died during the active study period. Follow-up survival data for all 181 patients who received the study drug were gathered through December 2009, at which time the median follow-up period was 54 weeks after the first dose of study drug. The 1-year overall survivals were 58.7% (95% CI, 48.4–69.1%) and 63.4% (95% CI, 53.4–73.3%) in the EPO and placebo groups, respectively (*P*=0.560, by the log-rank test), and the hazard ratio (HR) was 1.15 (95% CI, 0.72–1.85).

## Discussion

Erythropoietin-stimulating agents, one of the treatment options for anaemia, raise haemoglobin levels, reduce the proportion of patients requiring transfusions and improve QOL (Littlewood *et al*, 2001; Österborg *et al*, 2002; Boogaerts *et al*, 2003; Iconomou *et al*, 2003). However, recent meta-analyses on QOL have shown that ESAs induce a statistically significant but not clinically meaningful improvement of fatigue as measured with FACT-Fatigue (Tonelli *et al*, 2009; Minton *et al*, 2010). The ESAs have been approved for the treatment of CIA, and are widely used in the United States and Europe. The EPO is approved and marketed in Europe but not in the United States.

In recent years, however, several randomised clinical trials using ESAs (Hedenus *et al*, 2003; Henke *et al*, 2003; Leyland-Jones *et al*, 2005; Overgaard *et al*, 2007; Wright *et al*, 2007; Smith *et al*, 2008; Thomas *et al*, 2008) and meta-analyses (Bennett *et al*, 2008; Bohlius *et al*, 2009a, 2009b) have raised concerns about the negative impact on overall survival and tumour progression. Such safety issues regarding the use of ESAs in cancer patients have been discussed by regulatory authorities in the United States and Europe for several years (Juneja *et al*, 2008). To minimise the risks, both regulatory authorities have revised the labelling for ESAs and restricted their use in cancer patients. One of the restrictions in the United States is not to administer ESAs to patients with potentially curable cancers. Based on the decisions made by the EMA, the current labelling information specifies that ESAs should be administered to cancer patients with CIA whose haemoglobin level is ⩽10 g dl^–1^ and that a sustained haemoglobin level of >12 g dl^–1^ should be avoided. The present study was the first to evaluate the efficacy and safety of ESAs when dosed in accordance with the current labelling approved by the EMA in a randomised, double-blind, placebo-controlled manner. The inclusion criterion with regard to haemoglobin level was 8.0–10.0 g dl^–1^, and the median baseline haemoglobin level was 9.4 g dl^–1^ in the EPO group and 9.3 g dl^–1^ in the placebo group. If the haemoglobin level increased to >12.0 g dl^–1^ during the study period, the study drug was discontinued until the haemoglobin level decreased to ⩽11.0 g dl^–1^.

The results of this study demonstrated that once-weekly EPO administration significantly reduced the proportion of patients requiring RBC transfusions or having a haemoglobin level <8.0 g dl^–1^ after 4 weeks of treatment (10.0% *vs* 56.4%, *P*<0.001) and also reduced the proportion of patients requiring RBC transfusions (4.5% *vs* 19.6%, *P*=0.002); however, the dosing strategy in this study was conservative compared with those of previous studies (Boogaerts *et al*, 2003; Iconomou *et al*, 2003; Fujisaka *et al*, 2006; Morishima *et al*, 2006; Nakagawa *et al*, 2007; Aapro *et al*, 2008a, 2008b; Suzuki *et al*, 2008; Bohlius *et al*, 2009a, 2009b; Tsuboi *et al*, 2009). The relatively low percentage of patients receiving transfusions in both groups reflects the fact that most physicians hesitate to prescribe transfusions, preferring to monitor the situation until anaemia symptoms become remarkable. The pretransfusion haemoglobin levels at the time of the first transfusion in the current study were in the range of 5.3–8.1 g dl^–1^.

EPO was well tolerated in this study. The incidence and types of adverse events were similar between the EPO and placebo groups. Previous meta-analyses have indicated that the use of ESAs leads to an increased risk of thromboembolic events (relative risk (RR) 1.67; 95% CI, 1.35–2.06 (Bohlius *et al*, 2006) and RR 1.57; 95% CI, 1.31–1.87 (Bennett *et al*, 2008)). In the current study, one pulmonary embolism was observed during treatment with EPO, but no death due to thromboembolic events was reported.

The results of the latest Cochrane meta-analysis using individual patient data from 53 ESA trials were recently published in the *Lancet* (Bohlius *et al*, 2009a, 2009b). In this report, subgroup analysis of data from chemotherapy-treated patients (10 441 patients in 38 trials) indicated that the increase in mortality associated with ESAs was less pronounced in this population (HR for death during the active study periods=1.10; 95% CI, 0.98–1.24, *P*=0.12; HR for overall survival=1.04; 95% CI, 0.97–1.11, *P*=0.263) than in patients undergoing other anticancer treatments such as radiotherapy, radiochemotherapy or no anticancer treatment (HR 1.33–1.53). However, none of the studies included in the Cochrane meta-analysis used ESAs in accordance with the revised labelling indications (baseline haemoglobin levels, target and ceiling and so on). Although the current study was not designed and not powered to show that EPO did not increase mortality in this dosing scheme and that EPO was safe, the number of patients who died during the study period was one in the EPO group and none in the placebo group. The 1-year overall survival in the EPO group was 58.7% (95% CI 48.4–69.1%) and that in the placebo group was 63.4% (95% CI 53.4–73.3% log-rank, *P*=0.560). There have been considerable debates as to the mechanism by which ESAs increase the risk for mortality (Fandrey and Dicato, 2009). One possible explanation is that aggressive dosing with ESAs to achieve higher target haemoglobin levels (not recommended in the revised labelling information) can cause adverse effects. The FDA has requested that a prospective randomised controlled trial of the use of ESAs be carried out, assessing their safety at haemoglobin levels of <12 g dl^–1^. Such a trial is currently ongoing in patients with non-small cell lung cancer undergoing chemotherapy.

In conclusion, the findings from this study provide new evidence that ESAs are effective and well tolerated when used within the revised labelling indications by the EMA, with the limitation that we did not formally search for thromboembolic events. However, it is important that ESAs be used in accordance with the labelled indications. In addition, the risk of thromboembolic events and possible negative effects on survival should be carefully weighed against the benefits of ESA treatment in patients with CIA, taking into account the patients’ comorbidities and the conditions under which they are treated. Further investigations are needed on the effect of ESAs on mortality.

## Figures and Tables

**Table 1:** Characteristics of full analysis population

	**EPO (*n*=89)**	**Placebo (*n*=92)**
*Sex*		
Male	47	40
Female	42	52
		
Age (years), median (min–max)	67 (40–79)	63.5 (44–79)
Weight (kg), median (min–max)	53.5 (35–102)	52.8 (37.4–78.1)
		
*Tumour*		
Small cell lung cancer	20 (22.5)	22 (23.9)
Non-small cell lung cancer	40 (44.9)	38 (41.3)
Ovarian cancer	19 (21.3)	19 (20.7)
Other	10 (11.2)	13 (14.1)
		
*ECOG performance status*		
0	42 (47.2)	41 (44.6)
1	45 (50.6)	50 (54.3)
2	2 (2.2)	1 (1.1)
		
Haemoglobin (g dl^–1^), median (min–max)	9.4 (8.1–11.4)	9.3 (7.2–11.4)
Transferrin saturation (%), median (min–max)	25.1 (5.4–97.6)	24.1 (6.4–99.4)
Serum endogenous erythropoietin (mIU ml^–1^), median (min–max)	43 (7.78–577)	43.6 (10.5–320)

Abbreviations: EPO=epoetin-*β*; ECOG=Eastern Cooperative Oncology Group.

**Table 2:** Incidence of adverse events

	**EPO (*n*=89)**		**Placebo (*n*=92)**	
	**No. of patients**	**%**	**No. of patients**	**%**
Adverse events	88	98.9	92	100.0
				
*Common adverse events*				
Neutropenia	82	92.1	74	80.4
Leucopenia	81	91.0	77	83.7
Thrombocytopenia	61	68.5	55	59.8
Lymphocytopenia	44	49.4	52	56.5
Anorexia	43	48.3	50	54.3
Nausea	43	48.3	46	50.0
				
Adverse drug reactions	37	41.6	28	30.4
				
*Common adverse drug reactions*				
Constipation	6	6.7	2	2.2
Increased blood pressure	5	5.6	3	3.3
Diarrhoea	5	5.6	1	1.1

Abbreviation: EPO=epoetin-*β*.
